# Comment on ‘Survey on the Knowledge and Practices in Anorexia of Aging Diagnosis and Management in Japan’ by Takagi et al.

**DOI:** 10.1002/jcsm.13626

**Published:** 2024-10-20

**Authors:** Ying Cui

**Affiliations:** ^1^ Department of Public Health Science, Graduate School and Transdisciplinary Major in Learning Health Systems, Graduate School Korea University Seoul South Korea

To the Editor

I have read with great interest the article published in the *Journal of Cachexia, Sarcopenia and Muscle* on the knowledge and practices in anorexia of ageing (AA) diagnosis and management in Japan [1]. The study provides valuable insights into the current state of AA management among healthcare professionals in Japan, emphasizing the critical role of continuing education. While the article is well written and contributes meaningfully to the field, I believe there are several areas where constructive suggestions could further enhance the interpretation and application of the results.

From a statistical perspective, the study has some limitations that merit consideration. The use of descriptive statistics and chi‐square tests to compare differences between the education and non‐education groups is commendable. However, incorporating multivariable regression models could make the analysis even more insightful, as these models would effectively control for potential confounding factors such as participants' work experience, institutional resources and overall attitudes towards elderly care. Additionally, examining interaction effects could significantly enhance the persuasiveness of the study's findings. Education may impact the management of AA differently across various professions, regions or institutions. Considering these interaction effects within the statistical models would provide a deeper understanding of how these factors collectively influence the results [2].

To address the challenges highlighted by this study, I propose a multidisciplinary approach involving community health initiatives. A coordinated effort that includes healthcare providers, community health workers, social workers and government officials could create a more supportive environment for managing AA. For example, community‐based nutrition education programmes could be developed to reach a broader audience, including those outside academic settings. Additionally, leveraging the role of community health workers to monitor and manage AA in older adults could enhance early detection and intervention [3], particularly in underserved areas.

In conclusion, while the article offers valuable contributions to understanding AA management in Japan, our suggestions aim to make an already excellent article even better. I look forward to seeing continued efforts from healthcare professionals, volunteers, government officials and social workers to create a healthier and more supportive environment for older adults.

## Conflicts of Interest

The author received no specific funding for this work.

## Data Availability

The author has nothing to report.
